# Smallest [5,6]Fullerene as Building Blocks for 2D Networks with Superior Stability and Enhanced Photocatalytic Performance

**DOI:** 10.1021/jacs.4c13167

**Published:** 2024-11-19

**Authors:** Jiaqi Wu, Bo Peng

**Affiliations:** † Peterhouse, University of Cambridge, Trumpington Street, Cambridge CB2 1RD, UK; ‡ Theory of Condensed Matter Group, Cavendish Laboratory, University of Cambridge, J.J. Thomson Avenue, Cambridge CB3 0HE, UK

## Abstract

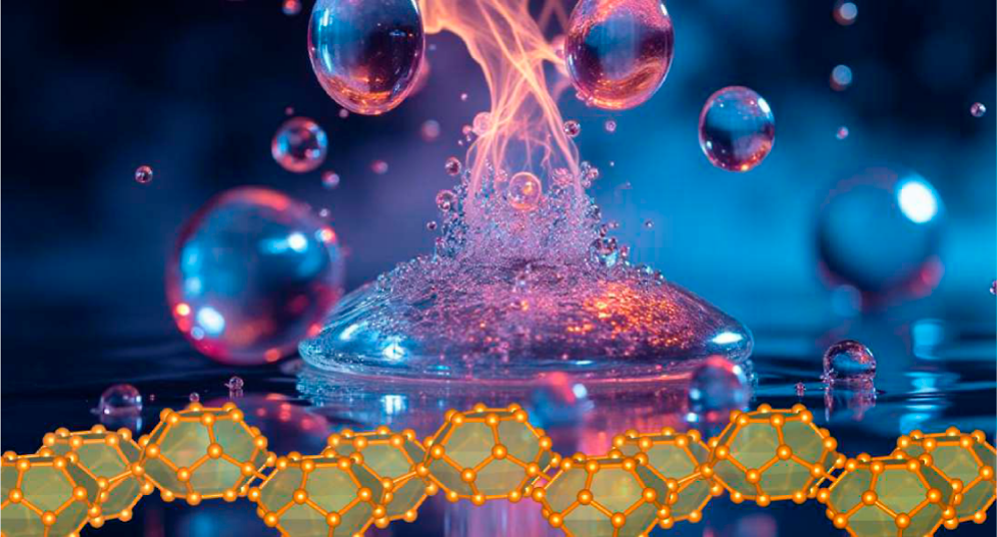

The assembly of molecules to form covalent networks can create varied lattice structures with physical and chemical properties distinct from those of conventional atomic lattices. Using the smallest stable [5,6]fullerene units as building blocks, various 2D C_24_ networks can be formed with superior stability and strength compared to the recently synthesized monolayer polymeric C_60_. Monolayer C_24_ harnesses the properties of both carbon crystals and fullerene molecules, such as stable chemical bonds, suitable band gaps, and large surface area, facilitating photocatalytic water splitting. The electronic band gaps of C_24_ are comparable to those of TiO_2_, providing appropriate band edges with sufficient external potential for overall water splitting over the acidic and neutral pH range. Upon photoexcitation, strong solar absorption enabled by strongly bound bright excitons can generate carriers effectively, while the type-II band alignment between C_24_ and other 2D monolayers can separate electrons and holes in individual layers simultaneously. Additionally, the number of surface-active sites of C_24_ monolayers are three times more than that of their C_60_ counterparts in a much wider pH range, providing spontaneous reaction pathways for the hydrogen evolution reaction. Our work provides insights into materials design using tunable building blocks of fullerene units with tailored functions for energy generation, conversion, and storage.

## Introduction

Carbon atoms provide building blocks for rich structural phases with a variety of physical and chemical properties .^1^ The sp^3^ hybridization of carbon atoms leads to cubic diamond, one of the hardest materials on Earth, while the covalently bonded sp^2^-hybridized carbon atoms in honeycomb layers can be held together by van der Waals interactions, resulting in the slipperiness of graphite. Instead of carbon atoms, fullerene molecules can form individual stable units that can be connected through intermolecular bonds, forming superatomic lattices beyond the conventional paradigm of atomic building blocks .^2−5^ Recently, multiple covalently bonded fullerene networks have been synthesized in 2D, namely, a quasi-tetragonal phase (qTP) and a quasi-hexagonal phase (qHP) in monolayer ^6^ and few-layer ^7,8^ forms. Such 2D polymeric fullerene is highly stable ^9−11^ with promising electronic and optical properties ^6,12,13^ for photocatalytic water splitting because of their suitable band gaps and abundant surface-active sites on large surface area, which has been predicted theoretically ^9,14^ and soon confirmed experimentally .^8^

Among all the fullerene clusters,^15−17^ the (C_24_*-D*_6d_)[5,6]fullerene cage represents the smallest stable conventional fullerene (with 5- and 6-membered rings) that has been predicted theoretically ^18,19^ and characterized experimentally in mass spectra from laser vaporization products of graphite .^20^ Such synthesis method facilitates fullerene growth through the stacking of C_6_ and C_12_ rings in laser-vaporized hot carbon soot,^21,22^ which has also been successfully applied in the synthesis of other fullerene cages such as C_36_ .^23^ The other approach utilizes a wide range of organic synthesis techniques to produce a hydrogenated carbon cage,^24,25^ followed by bromine substitution and gas-phase debromination, which has also been used in the production of fullerene C_20_ .^26^ Moreover, C_24_ has been found to be a plausible carrier for the 11.2 μm unidentified infrared band in many different galactic and extragalactic environments .^27^ The highly symmetric molecular structure of C_2__4_, in combination with the rich variety of carbon–carbon bonds, allows the formation of various monolayer networks of polymeric C_2__4_ similar to polymeric C_60_. However, it is unclear whether the change of molecular size in such superatomic lattices can tune the physical and chemical properties of monolayer polymeric fullerene in terms of structural stability, electronic structures, optical absorption, and chemical reactivity on the surface.

In this work, we find that the C_24_ molecules are energetically more favorable to form monolayer networks than C_60_. Additionally, these C_24_ monolayers are more promising as highly stable photocatalysts for overall water splitting. Compared to monolayer polymeric C_60_, monolayer C_2__4_ networks exhibit superior thermodynamic, dynamic, and mechanical stability, indicating the experimental feasibility in synthesizing such monolayers. Our hybrid functional calculations show that monolayer phases of C_24_ have band gaps comparable to those of TiO_2_, with suitable band gaps to drive overall water splitting over the entire acidic pH range. Most interestingly, while bright excitons near the band edges lead to strong absorption in the solar spectrum for efficient carrier generation, the electron–hole pairs can be effectively separated into individual layers by combining C_24_ with other monolayers in type-II van der Waals heterostructures. Moreover, the large surface area of C_24_ monolayers provides abundant surface-active sites to drive the reactions spontaneously in multiple catalytic pathways, with over triple the number of adsorption sites compared to C_60_ monolayers at pH > 3.

## Methods

Density functional theory (DFT) calculations are performed using the Vienna Ab initio Simulation Package (VASP) .^28,29^ The projector-augmented wave (PAW) basis set ^30,31^ is used for C valence states of 2s^2^2p^2^ under the generalized gradient approximation (GGA) formalism using the Perdew–Burke–Ernzerhof functional revised for solids (PBEsol) .^32^ A plane-wave cutoff of 800 eV is used with the Brillouin zone sampled by a **k**-mesh of 5 × 5 and 3 × 5 for qTP and qHP C_24_ monolayers, respectively. The lattice constants and atomic coordinates are fully relaxed using the energy and force convergence criteria of 10^–6^ eV and 10^–2^ eV/Å, respectively. The interlayer spacing is larger than 23 Å, and dipole corrections are applied^33^ in order to eliminate the electrostatic interactions across periodic images along *z*. For isolated C_24_ molecules and C atoms, the spacing in all directions is larger than 29 Å, and a **k**-mesh of 1 × 1 × 1 is employed. The atomic coordinates of the C_24_ molecules are fully relaxed with the same energy and force convergence criteria as those above.

The phonon spectrum is calculated under the harmonic approximation using phonopy ^34,35^ with interatomic force constants computed from density functional perturbation theory (DFPT) .^36,37^ A supercell size of 2 × 2 and a **k**-mesh of 3 × 3 are used for both qTP and qHP C_24_. The Helmhotz free energy of the monolayers is evaluated by summing on an 81 × 81 phonon **q**-mesh and adding electronic contributions. Elastic constants of the monolayers are obtained using the finite difference method .^38,39^

The electronic structures of the systems are calculated with the hybrid Heyd–Scuseria–Ernzerhof functional revised for solids (HSEsol) ^40^ with a screening parameter μ = 0.2 Å^–1^ ^41−43^ and PBEsol mixed with unscreened exact Hartree–Fock exchange (PBEsol0) .^44^ It has previously been shown that unscreened hybrid functional PBEsol0 describes best the band gaps of monolayer fullerene networks ^9,14^ compared to the measured ones ^6,8^ and the results from the computationally heavy many-body perturbation theory .^45^

Excitonic effects are calculated from the time-dependent Hartree–Fock (TDHF) method using the Casida equation ^46^ on top of the PBEsol0 eigenenergies and wave functions. Previous work has shown that this method yields quantitative agreements ^9,14^ with the Bethe–Salpeter equation (BSE) on top of many-body perturbation theory .^45^ The Tamm–Dancoff approximation is used as the discrepancy is within 5 meV .^47^ A basis of 16 highest valence bands and 16 lowest conduction bands is used with a **k**-mesh of 10 × 10 and 6 × 10 for qTP and qHP, respectively. The exciton eigenenergies and optical absorption curves are well converged. The dimensionless absorbance in 2D materials is defined as
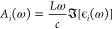
1where ϵ_*i*_(ω) is the complex dielectric function along polarization direction *i* at photon frequency ω, *c* is the speed of light, and *L* is the interlayer distance.

The thermodynamics at different active sites are calculated by placing one hydrogen atom near each symmetry-irreducible carbon atom and then performing full relaxation in a supercell of 3 × 3 (qTP) and 2 × 3 (qHP) to ensure a minimum of 15 Å between neighboring adsorbates. Thermal corrections at *T* = 300 K, *p* = 1 atm are added to the electronic free energy by
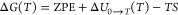
where ZPE is the zero-point energy, *U* is the internal energy, and *S* is the entropy, with an extra pV term for gas molecules. Under the standard hydrogen electrode approximation, the free energy of 1/2 H_2_ and *e*^−^ + H^+^ are equal at equilibrium, while the external potential of photoexcited electrons is taken as the difference between the conduction band minimum and the hydrogen evolution reaction (HER) potential .^48,49^

## Results and Discussion

### Crystal Structures

**Figure 1:**
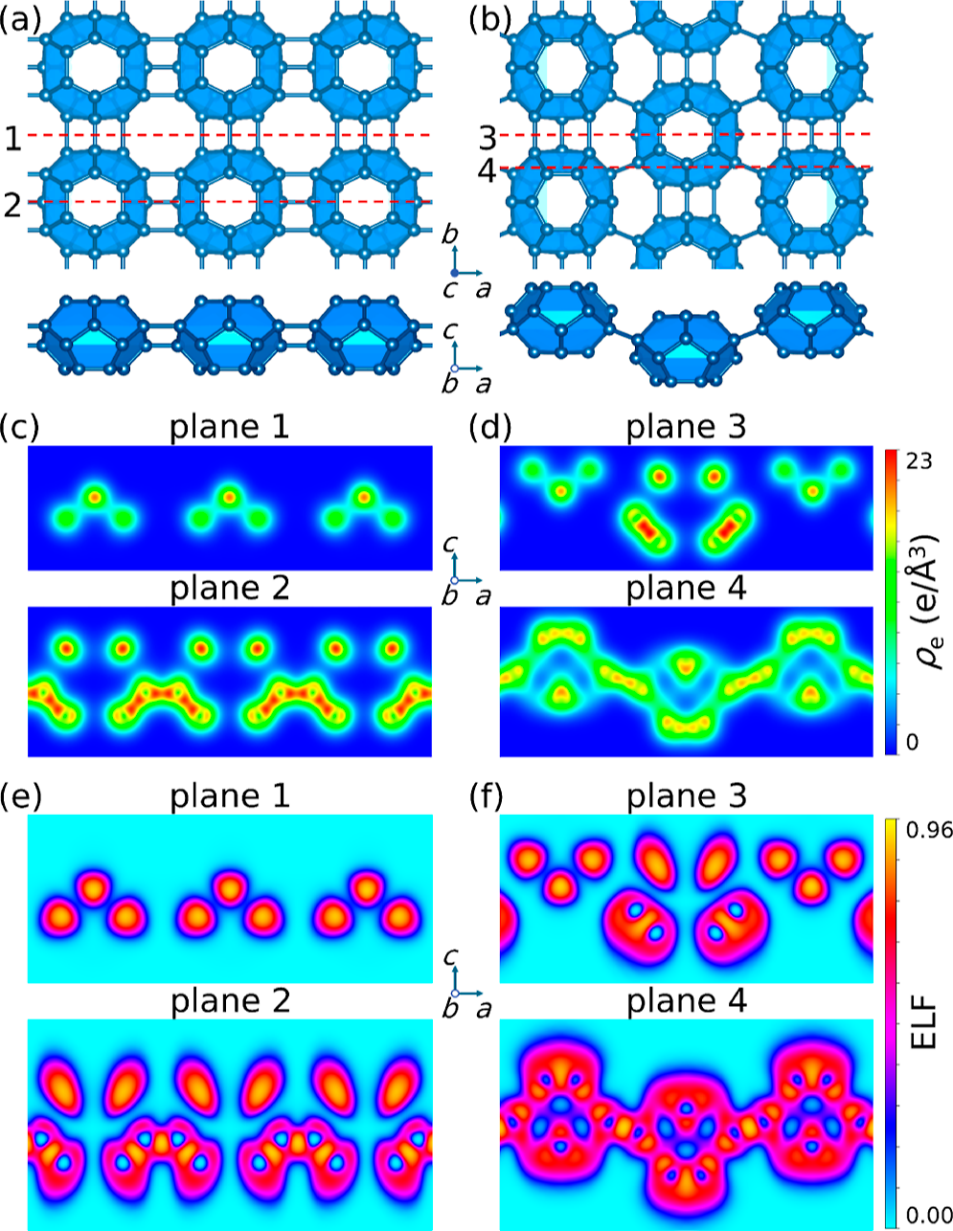
Top and side views of crystal structures of (a) qTP and (b) qHP C_24_ monolayers. Charge density on selected (010) planes plotted by VESTA ^50^ showing the intermolecular bonding features of (c) qTP and (d) qHP C_24_ monolayers, as well as their corresponding ELF for (e) qTP and (f) qHP C_24_ monolayers.

The crystal structures of the qTP and qHP monolayers are presented in Figure 1a,b. The overall structure for C_24_ qTP monolayer is analogous to the C_60_ equivalent and can be regarded as a nearly square lattice with intermolecular bonds between neighboring C_24_ units. One difference between qTP C_24_ and C_60_ monolayers is that the lattice constants *a* and *b* are equal for C_24_ but slightly different for C_60_ (Table 1) because of different molecular symmetry. Moreover, due to the different molecular geometry, qTP C_24_ monolayers exhibit three noncoplanar bonds between neighboring C_24_ clusters, instead of the [2 + 2] cycloaddition bonds for qTP C_60_. In addition, the central bond is slightly shorter than the two side bonds, leading to a higher charge density, as shown on plane 1 in Figure 1c. This suggests a stronger cohesive interaction relative to their C_60_ counterparts.

**Table 1:** Lattice Constants, Cohesive Energies per Atom *E*_c_, and Additional Cohesive Energy for Monolayer Formation from Molecules Δ*E*_c_ of C_24_ Phases Calculated from PBEsol^a^

phase	*a* (Å)	*b* (Å)	*E*_c_ (eV)	Δ*E*_c_ (eV)
0D	/	/	–8.586	0.000
	/	/	(−9.256)	(0.000)
2D qTP	6.103	6.103	–8.914	–0.328
	(9.097)	(9.001)	(−9.259)	(−0.002)
2D qHP	11.500	6.180	–8.974	–0.388
	(15.848)	(9.131)	(−9.246)	(+0.010)

a The values for *C*_60_ are shown in parentheses for comparison.

The qHP C_24_ monolayer can be interpreted as misaligned 1D chains along the *b* direction connected by the three noncoplanar bonds, which are further joined through diagonal single bonds between neighboring chains along *a*. The monolayer exhibits a buckled structure owing to the asymmetry of the interchain bonding positions. The charge density plots on plane 3 in Figure 1d show that the three noncoplanar bonds along *b* are similar to the qTP bonds, while the diagonal bonds are slightly weaker. This leads to mechanical anisotropy, as discussed later. We also investigate the electron localization function (ELF) .^51−53^ ELF = 1 corresponding to perfect localization and ELF = 0.5 corresponding to the electron-gas-like pair probability. The ELF plots in Figure 1e,f show large areas of ELF = 0.5 around the C_2__4_ units. This indicates the diffuse, delocalized π features of the C_24_ cage.

The cohesive energy per atom is defined as
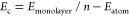
2where *E*_monolayer_ is the energy of the monolayer per unit cell, *n* is the number of carbon atoms in the unit cell, and *E*_atom_ is the energy of an isolated carbon atom. As summarized in Table 1, both monolayers are more stable with respect to isolated C_24_ molecules by 0.328 eV/atom for qTP and 0.388 eV/atom for qHP. Monolayer qHP C_2__4_ is energetically more favorable by 0.060 eV/atom possibly due to its close-packed structure. In comparison, the additional cohesive energy for the formation of monolayer C_60_ networks is much weaker, with −0.002 eV for qTP and (endothermic) +0.010 eV for qHP. This can be rationalized by the release of stereochemical strain in C_24_ units through forming sp^3^-like sites at the non-coplanar bonds, suggesting energetically more favorable formation of C_24_ monolayers from molecules.

### Stability

**Figure 2:**
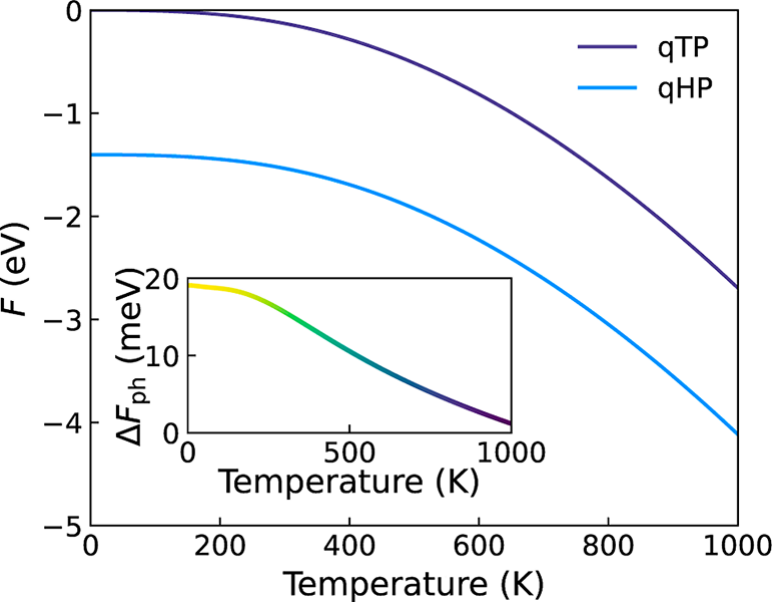
Free energy curves of qHP and qTP phases per C_24_ unit, with the free energy of qTP at 0 K set to zero. The phonon free energy difference between qTP and qHP (Δ*F*_ph_) is shown in the inset.

**Figure 3:**
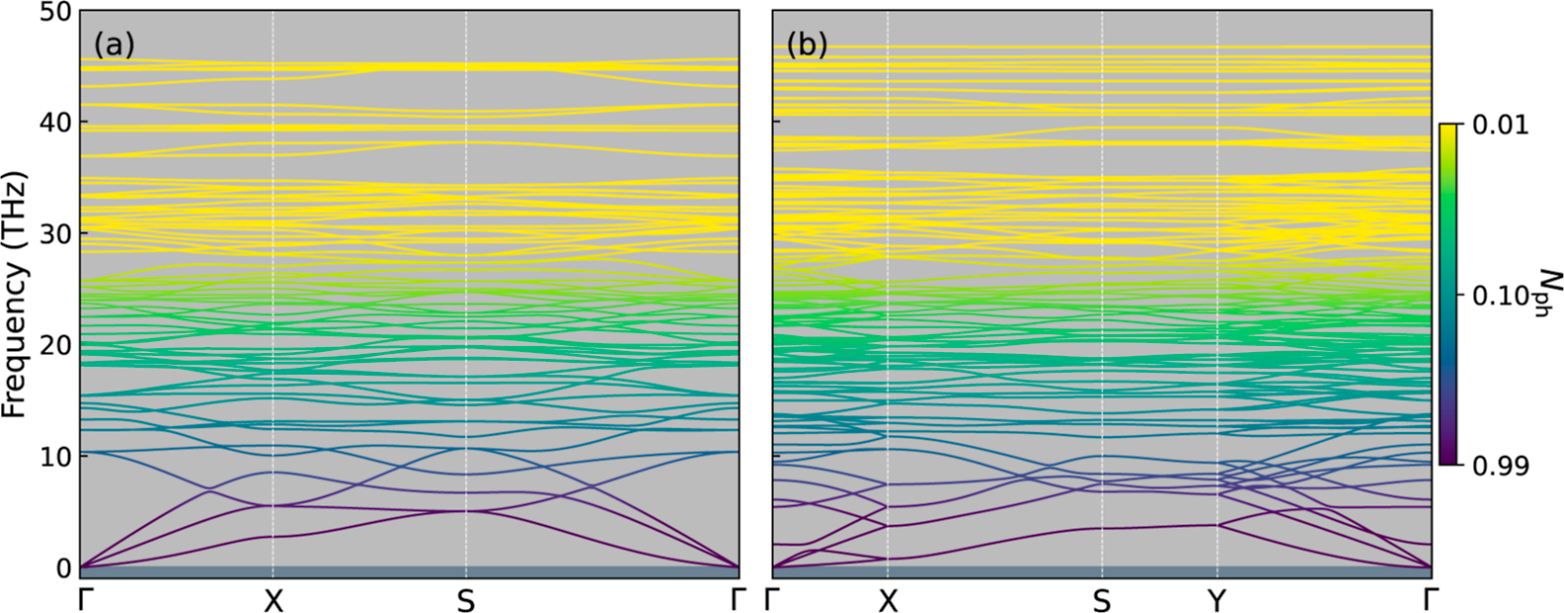
Phonon spectra of (a) qTP and (b) qHP C_24_ monolayers, with the phonon occupation number *N*_ph_ from the Bose–Einstein distribution at 300 K.

The thermodynamic stability of the monolayers is analyzed by considering electronic and vibrational free energies. The variation of total free energy *F* with temperature is shown in Figure 2. The temperature dependence in *F* is due to the thermal activation of phonon modes shown by the Bose–Einstein phonon occupation number in Figure 3, while the approximately constant difference of total free energy between qTP and qHP is dominated by the electronic free energy difference of Δ*F*_el_ = 1.42 eV. The phonon free energy for qHP is slightly higher at low temperatures due to the zero-point energy of more high-frequency phonon modes, and the difference Δ*F*_ph_ approaches zero around 1000 K. The thermodynamically stable phase at all temperatures of interest is qHP, in contrast to the results for C_60_ monolayers .^10^

To evaluate the dynamic stability of the C_24_ monolayers, the phonon dispersion curves are investigated in Figure 3. Near Γ, both dispersion curves exhibit two Debye-like linear acoustic modes corresponding to the in-plane longitudinal and transverse modes. The additional quadratic acoustic mode corresponds to an out-of-plane flexural mode, which is a characteristic of monolayer structures. Both phases exhibit no imaginary-frequency phonon mode, indicating that the system is at an energy minimum on the potential energy surface .^54−56^ The dynamic stability is a sign of the strong covalent bonding of the C_24_ monolayers.

We next investigate the mechanical stability of the two monolayer phases. The 2D elastic constants (in Voigt notation) are normalized from the 3D lattice constants by
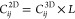
3where *L* is the interlayer separation. The elastic constants are summarized in Table 2. The Born–Huang dynamical lattice theory sets the criteria of mechanical stability for qTP (space group *P*4̅*m*2)
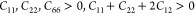
4and an additional criterion for qHP (space group *Pmmn*)
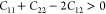
5

**Table 2:** Elastic Constants *C*_*ij*_, Layer Moduli γ, Young’s Moduli *Y*^2D^, and Shear Moduli *G*^2D^ (N/m) of qTP and qHP

phase	*C*_11_	*C*_22_	*C*_12_	*C*_66_ = *G*^2D^	γ	*Y*_*a*_^2D^	*Y*_*b*_^2D^
qTP	239.9	239.9	–6.2	78.2	116.8	239.7	239.7
qHP	221.3	268.9	20.6	103.4	132.8	219.7	267.0

Both qTP and qHP fit these criteria, indicating good mechanical stability. The elastic moduli can be calculated using
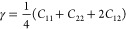
6
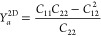
7
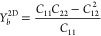
8
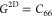
9where γ is the layer modulus (the 2D equivalent of the bulk modulus), and *Y*^2D^ and *G*^2D^ are the 2D Young’s and shear moduli, respectively. Monolayer qTP C_24_ is elastically isotropic along the *a* and *b* directions due to its *S*_4_ (4-fold improper rotation) symmetry. In monolayer qHP C_2__4_, *Y*_*a*_^2D^ is smaller than *Y*_*b*_^2D^, in agreement with the weaker diagonal interchain single bonds. In comparison, qHP C_2__4_ has a larger γ and *Y*_*b*_^2D^ than qTP due to the more close-packed structure and higher concentration of interfullerene bonds. The shear modulus *G*^2D^ of qHP is much larger than that of qTP due to the difficulty of dislocating the close-packed misaligned chains. Interestingly, the Young’s and layer moduli of both C_2__4_ monolayers are about 1.5 times higher than those of their C_60_ counterparts .^10^ This is rationalized by the presence of triple noncoplanar bonds in C_2__4_ monolayers, compared with the [2 + 2] cycloaddition bonds of C_60_ monolayers. Another contributing factor is the smaller molecular size and hence higher density of interfullerene bonds.

Given its superior stability and strength compared to that of monolayer polymeric C_60_, it is plausible that synthesis and exfoliation of the C_2__4_ monolayers are experimentally more feasible. We next explore the photocatalytic properties of monolayer C_2__4_ because of their large surface area for maximum contact with aqueous species for reaction. The crucial requirements for a photocatalyst are (i) a suitable band edge for driving the reaction, (ii) strong optical absorbance to generate photoexcited carriers effectively, and (iii) abundant surface-active sites, which will be discussed in the next three subsections.

### Electronic Structures

**Figure 4:**
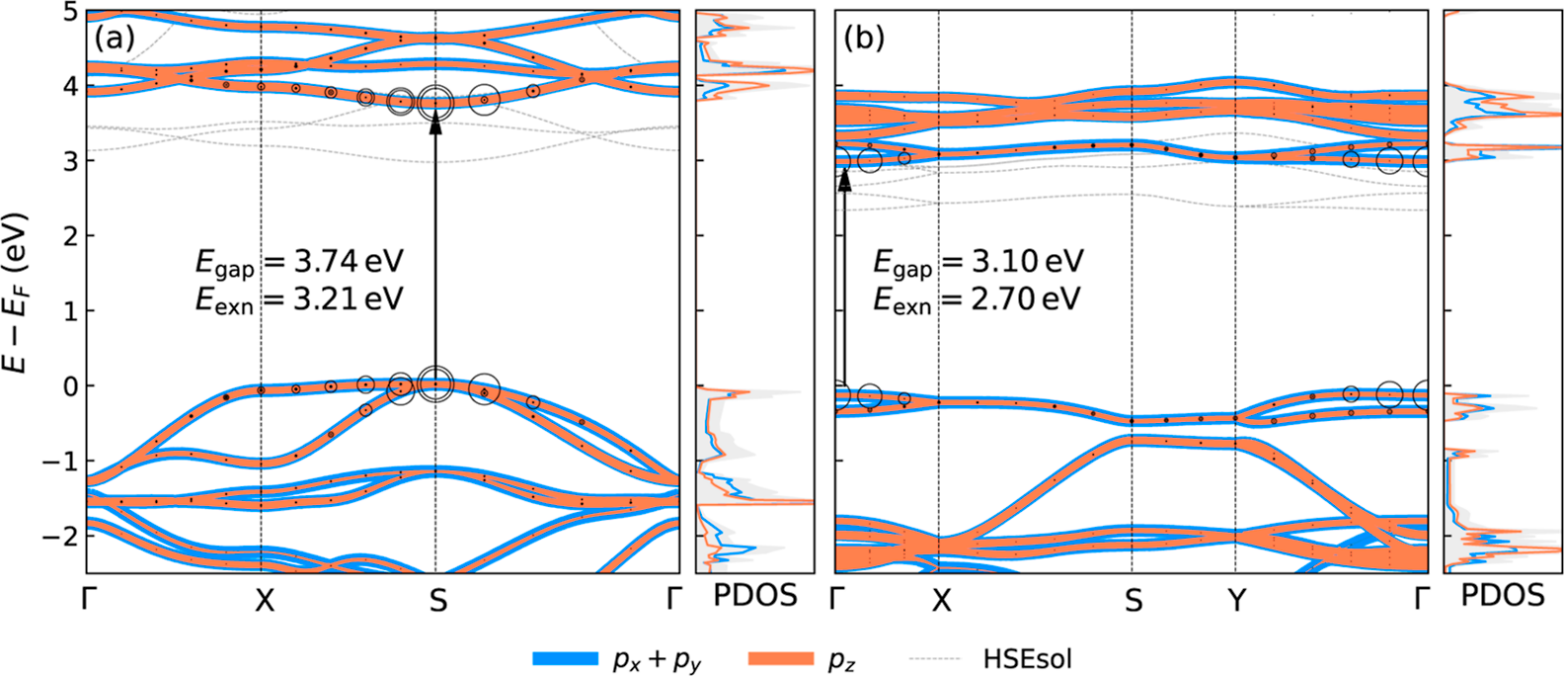
Orbital-projected band structures with projected density of states (PDOS) for (a) qTP and (b) qHP calculated at the PBEsol0 level. The HSEsol band structures are shown in light gray for comparison. The electron–hole pairs that contribute to the first bright exciton are also shown, with larger circles indicating more contributions from the electron–hole pairs at a given **k**.

Figure 4 shows the band structure of qTP and qHP C_2__4_ monolayers calculated from unscreened hybrid functional PBEsol0. PBEsol0 predicts the most accurate band gap for monolayer and few-layer polymeric C_60_ ^9,14^ that is in good agreement with the measured band gap ^6,8^ and with the many-body perturbation theory;^45^ hence, we focus on the PBEsol0 band edges hereafter. Monolayer qTP C_24_ has a direct band gap of 3.74 eV at the S high-symmetry point. Monolayer qHP C_24_ has an indirect band gap of 3.10 eV, with the valence band maximum (VBM) around the middle of ΓY and the conduction band minimum (CBM) at Γ. However, the energy difference between the VBM and the highest valence band at Γ is less than 14 meV, leading to a direct-like characteristic. Interestingly, the PBEsol0 band gaps of qTP and qHP C_24_ monolayers are comparable to those of TiO_2_, the most widely used oxide for photocatalytic applications .^57−67^ As a comparison, HSEsol leads to smaller band gaps of 3.03 and 2.42 eV for qTP and qHP C_24_, respectively, providing the lower bound of the band gaps. The HSEsol valence bands overlap with the PBEsol0 bands when the Fermi energy is set to zero, while the HSEsol conduction bands are a rigid shift of the PBEsol0 bands.

**Figure 5:**
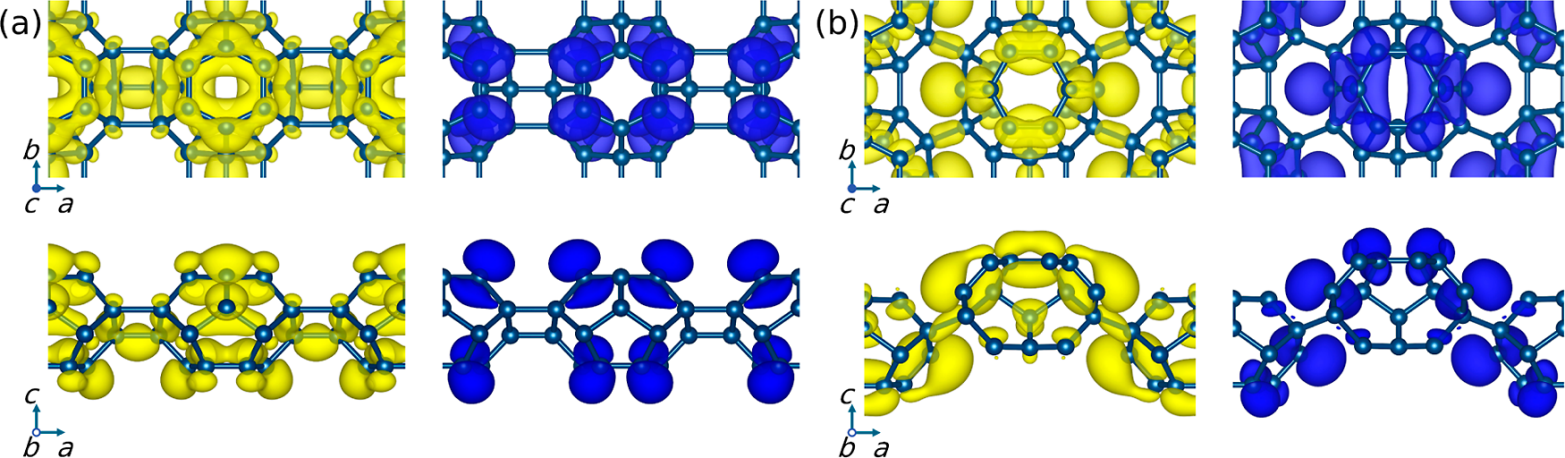
Top and side views of the VBM (yellow) and CBM (navy) charge densities with the largest contribution to the first bright exciton for (a) qTP and (b) qHP C_24_. The VBM of qTP C_24_ corresponds to a superposition of doubly degenerate states.

The band edges are mainly contributed to by the 2p orbitals of carbon. The p_*z*_ orbitals consist of half the DOS at the band edge, resulting in highly delocalized states. Figure 5a,b shows the band edge states of qTP and qHP, respectively. The VBM states of qTP are doubly degenerate at S with significant contributions from the π orbitals from the top and bottom hexagonal rings of C_24_ units. The CBM of qTP is a combination of two π* orbitals from the top hexagonal ring and two π* orbitals from the bottom hexagonal ring, in line with the almost full p_*z*_ character. For qHP, the partial charge density mainly comes from the π and π* orbitals of the hexagonal ring on the outward side of the buckled structure for both VBM and CBM. The delocalized band edge states on the top and bottom hexagonal rings can be promising for stereochemical interactions between the catalyst and aqueous species.

**Figure 6:**
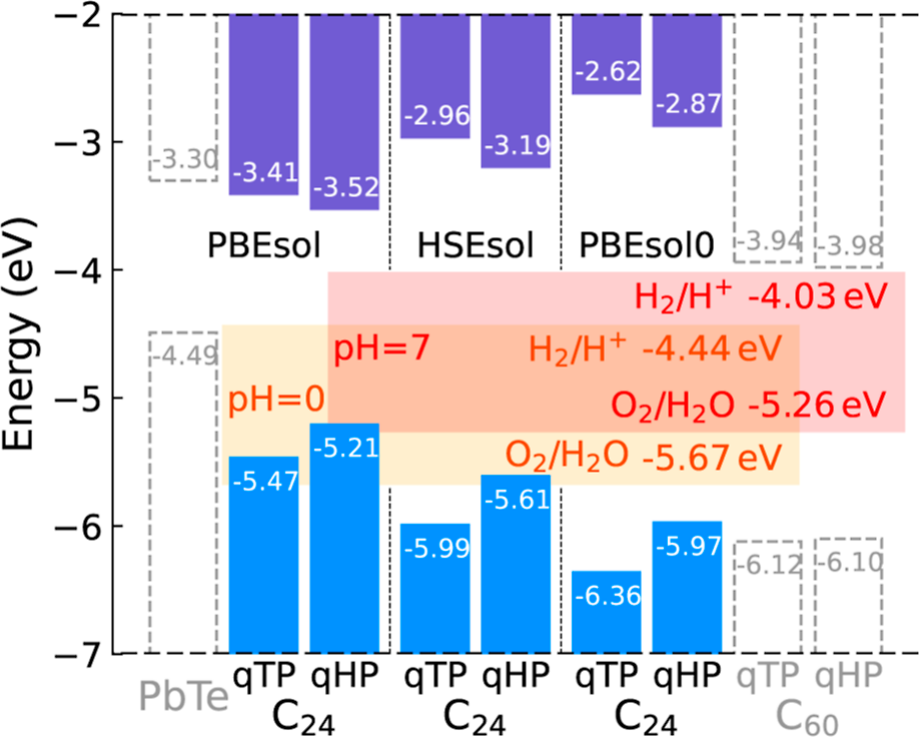
Band edges of monolayer qTP and qHP C_24_ with respect to the vacuum level calculated from PBEsol, HSEsol, and PBEsol0 compared with the redox (HER/OER) potentials of water at pH = 0 and 7. The purple and blue rectangles represent the CBM and VBM, respectively.

For a water-splitting photocatalyst, the band edges need to straddle the redox potentials of water; i.e., the VBM must be lower than the oxygen evolution reaction (OER) potential of water, −5.67 + pH × 0.059 eV, and the CBM must be higher than the HER potential of water, −4.44 + pH × 0.059 eV. Figure 6 shows the VBM and CBM calculated from PBEsol, HSEsol, and PBEsol0, with the vacuum level set to zero under the absolute electrode potential model. The redox potentials of water are shown as horizontal rectangles at pH = 0 and 7.

Benefiting from the large band gaps, both qTP and qHP C_24_ fit the photocatalysis criteria quite well, even at the PBEsol level in a wide pH range. We hereafter focus on the PBEsol0 band edges because unscreened hybrid functional is more accurate in describing systems with weak screening .^68^ For qTP C_24_, the VBM is 0.69 eV below the OER potential at pH = 0 and the CBM is 1.41 eV above the HER potential at pH = 7. For qHP C_24_, the VBM is 0.30 eV below the pH = 0 OER potential and the CBM is 1.16 eV above the pH = 7 HER potential. Therefore, both qTP and qHP C_24_ can be promising photocatalysts in a wide pH range from acidic to neutral pH. In contrast, C_60_ monolayers have much smaller band gaps, with the CBM almost touching the HER potential at pH = 7 .^9^ This suggests enhanced photocatalytic activity of C_24_ monolayers compared to that of monolayer C_60_ networks.

### Optical Properties

**Figure 7:**
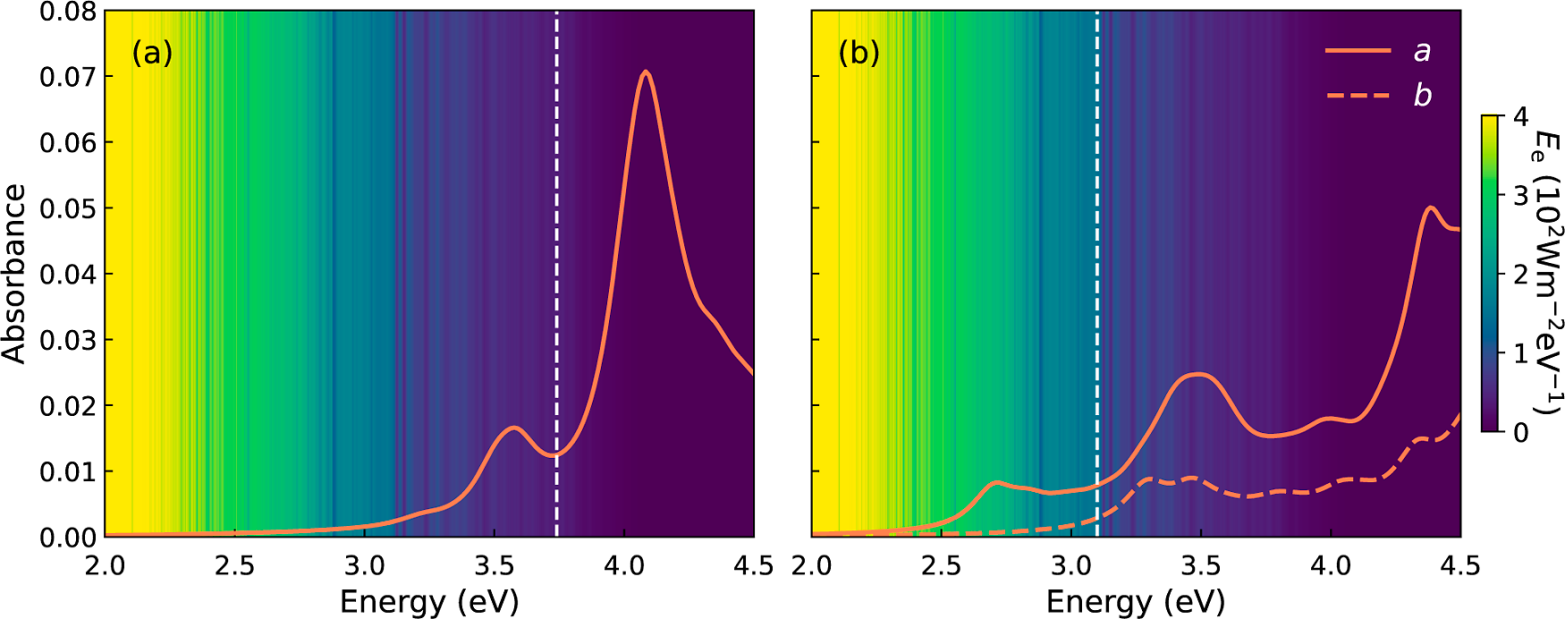
Excitonic absorbance of (a) qTP and (b) qHP C_24_ monolayers with the band gaps shown as the white lines. For qHP C_24_, the solid and dashed lines correspond to the *a* and *b* polarization directions. The background shows the global total spectral irradiance *E*_e_ from the Sun.

We then consider optical absorbance of C_24_ monolayers to investigate whether enough photoexcited carriers can be generated under the solar spectrum. Figure 7 shows the exciton absorbance of C_24_ monolayers computed from PBEsol0 + TDHF. Both phases exhibit a strong exciton absorption below the band gap (white dashed line) due to the direct nature of band gap and suitable molecular symmetry for optical transition selection rules. For qTP C_24_, the first bright exciton has an eigenenergy of 3.21 eV with an exciton binding energy of 0.53 eV, which is contributed mainly by the VBM and CBM states at S, as shown by the exciton fatband in Figure 4a. Even brighter excitons with higher oscillator strengths at 3.50 and 3.59 eV result in strong absorption peaks in the long-wavelength ultraviolet (UV-A) region.

For qHP, the absorbance is highly anisotropic along *a* and *b*. There is a strong exciton absorption peak above 2.5 eV along *a*, corresponding to the first bright exciton of 2.70 eV with a binding energy of 0.40 eV consisting of the VBM and CBM states at Γ. The absorbance along *b* is much weaker than that along *a* below the band gap. Above the band gap, strong absorbance peaks are found at around 3.5 eV along both directions, which further enhance UV-A absorption.

To separate electrons and holes effectively, C_24_ monolayers can be combined with other 2D materials to form van der Waals heterostructures with a type-II band alignment. As an example, monolayer PbTe in a 4 × 4 supercell only has a slight lattice mismatch below 0.5% with qTP C_24_ in a 3 × 3 supercell, and their band edges form the type-II alignment (Figure 6). On the other hand, the type-I band alignment between qTP and qHP monolayers, in combination with the strongly bound excitons with large oscillator strength and high binding energy, can be utilized in light-emitting devices owing to high emission efficiency .^69^

### Surface-Active Sites

**Figure 8:**
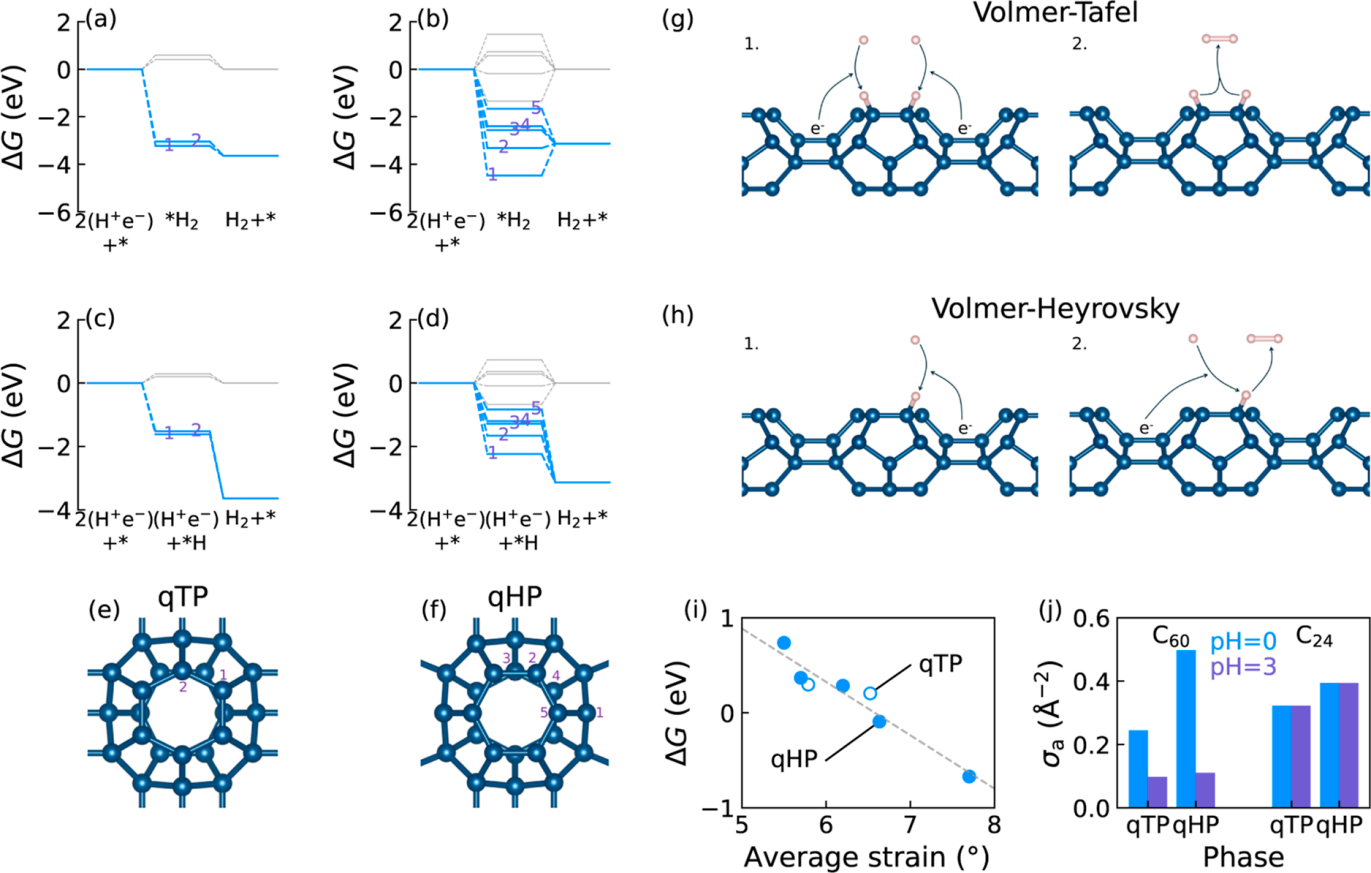
Free energy profiles of hydrogen evolution at different adsorption sites through the Volmer-Tafel mechanism for (a) qTP and (b) qHP C_24_ and the Volmer-Heyrovsky mechanism for (c) qTP and (d) qHP C_24_ at pH = 0, with gray and blue lines representing the absence and presence of photoexcitation, respectively. Symmetry-irreducible adsorption sites for (e) qTP and (f) qHP C_24_. (g) Volmer-Tafel and (h) Volmer-Heyrovsky reaction mechanisms. (i) Correlation between adsorption free energy and average bond angle strain. (j) Area density *σ*_a_ of active sites for various phases of fullerene monolayers at changing pH.

The thermodynamic driving force for water splitting is investigated by calculating the free energy of the hydrogen-adsorbed monolayers. Figure 8a–d shows the free energy pathways of HER at pH = 0. After full relaxation, hydrogen atoms are all adsorbed at the top sites, except for the chemically saturated atoms on the interfullerene bonds. All symmetry-irreducible adsorption sites of H^+^ ions on qTP and qHP monolayers are listed in Figure 8e,f, respectively, numbered from low to high free energy. Due to the buckled structure of qHP, the sites on the convex (sites 2, 5) and concave (sites 3, 4) surfaces are inequivalent with different free energies of reaction intermediates.

The HER has two possible catalytic pathways:^70^ (1) two protons are individually adsorbed onto the monolayer first and then chemically desorb by combining into a hydrogen molecule (Volmer-Tafel, V-T) (see Figure 8g); and (2) a single proton is adsorbed first, and a second proton approaches the adsorbed proton to form an electrochemically desorbed hydrogen molecule (Volmer-Heyrovsky, V-H) (see Figure 8h). The reaction scheme is summarized as
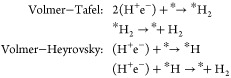
where * is the catalyst, i.e., the C_24_ monolayer, and (H^+^e^–^) denotes a pair of an aqueous proton in the solution and a photoexcited electron in the monolayer.

The thermodynamic requirement for high reaction rates is that the free energy must decrease along each step of the reaction pathway. Among all the reaction intermediates of qTP through both the V-T and V-H mechanisms, even the lowest free energy barrier in Figure 8a,c is still significantly larger than the thermal fluctuation energy *k*_*B*_*T* at room temperature (0.026 eV) without photoexcitation. With photoexcitation, HER occurs spontaneously at all adsorption sites of qTP C_24_ at pH = 0 along both the V-T and V-H pathways, indicating promising photocatalytic efficiency. For qHP C_24_, sites 1 and 2 are not active for the V-T mechanism because the free energies of their intermediates are lower than that of the final product, as shown in Figure 8b. However, the HER at these two sites can still proceed simultaneously via the V-H route in Figure 8. Furthermore, even up to neutral pH, the reactions at all available adsorption sites for both qTP and qHP monolayers are still spontaneous for at least one of the mechanisms.

To investigate the microscopic mechanism of adsorption free energy, we compare the free energy at all sites with the bond angle strain, which is defined as the difference between the average of the three bond angles at each site and the standard sp^2^ of 120°. The bond angle strain represents the deviation of the local carbon environment from an ideal flat sp^2^ surface, i.e., graphene. As shown in Figure 8i, the adsorption free energy at all sites exhibits a strong correlation with the bond angle strain. This implies that the local bond environment has a significant influence on hydrogen adsorption, especially for the small C_24_ fullerene with relatively higher curvature compared to C_60_.

Figure 8j summarizes the area density *σ*_a_ of thermodynamically active sites for qTP and qHP monolayers. Monolayer qHP C_24_ has more surface-active sites than qTP C_24_ due to the presence of the additional site 1 on the single-bond sides of the C_24_ units. At pH = 0, C_24_ monolayers have comparable active site densities to those of C_60_ monolayers. However, the number of active sites of C_60_ monolayers decrease with increasing pH. Even at a moderate pH = 3, the majority of the sites in C_60_ monolayers are no longer active, while all the active sites in C_24_ monolayers remain. Consequently, the number of surface-active sites in C_24_ monolayers is tripled compared to that in C_60_ monolayers at pH = 3, and the ratio continues to increase with higher pH.

## Conclusions

Using the smallest stable conventional [5,6]fullerene cage as building blocks, we predict two phases of monolayer C_24_ networks with superior stability and strength compared to that of their C_60_ counterparts, indicating high feasibility in synthesizing such monolayers with strong resilience to ambient temperatures and mechanical deformation. The band gaps of these C_24_ monolayers are much larger than that of their C_60_ counterparts while comparable to TiO_2_, providing suitable band edges for photocatalytic water splitting in a wide pH range from 0 to 7. Additionally, both phases have strong optical absorption benefiting from multiple bright excitons from the visible to UV-A range, enabling effective generation of a large amount of photoexcited carriers. Compared to monolayer polymeric C_60_, the density of surface-active sites are tripled in C_24_ monolayers. These results indicate that the photocatalytic performance of the C_24_ monolayers can be significantly enhanced. Beyond photocatalysis, we demonstrate the possibility of tuning physical and chemical properties of carbon nanomaterials by using different fullerene building blocks. Given the rich family of currently known fullerene molecules, new 2D materials can be designed with tunable and tailored functions.
